# The effect of the TLR9 ligand CpG-oligodeoxynucleotide on the protective immune response to alcelaphine herpesvirus-1-mediated malignant catarrhal fever in cattle

**DOI:** 10.1186/1297-9716-45-59

**Published:** 2014-05-27

**Authors:** Nevi Parameswaran, George C Russell, Kathryn Bartley, Dawn M Grant, David Deane, Helen Todd, Mark P Dagleish, David M Haig

**Affiliations:** 1 School of Veterinary Medicine and Science, University of Nottingham, Sutton Bonington, LE12 5RD Nottingham, UK; 2 Moredun Research Institute, Pentland Science Park, Bush Loan, EH26 0PZ Penicuik, UK

## Abstract

We wished to determine the effect of of CpG ODN adjuvant on the magnitude and duration of protective immunity against alcelaphine herpesvirus-1 (AlHV-1) malignant catarrhal fever (MCF), a fatal lymphoproliferative disease of cattle. Immunity was associated with a mucosal barrier of virus-neutralising antibody. The results showed that CpG ODN included either with emulsigen adjuvant and attenuated AlHV-1 (atAlHV-1) or alone with atAlHV-1 did not affect the overall protection from clinical disease or duration of immunity achieved using emulsigen and atAlHV-1. This is in contrast to other similar studies in cattle with BoHV-1 or cattle and pigs with various other immunogens. In addition to this, several other novel observations were made, not reported previously. Firstly, we were able to statistically verify that vaccine protection against MCF was associated with virus-neutralising antibodies (nAbs) in nasal secretions but was not associated with antibodies in blood plasma, nor with total virus-specific antibody (tAb) titres in either nasal secretions or blood plasma. Furthermore, CpG ODN alone as adjuvant did not support the generation of virus-neutralising antibodies. Secondly, there was a significant boost in tAb in animals with MCF comparing titres before and after challenge. This was not seen with protected animals. Finally, there was a strong IFN-γ response in animals with emulsigen and atAlHV-1 immunisation, as measured by IFN-γ secreting PBMC in culture (and a lack of IL-4) that was not affected by the inclusion of CpG ODN. This suggests that nAbs at the oro-nasal-pharyngeal region are important in protection against AlHV-1 MCF.

## Introduction

Malignant catarrhal fever (MCF) is a usually-fatal lymphoproliferative disease of a range of ungulates including cattle, deer, bison, water buffalo and pigs [1]. It is caused by members of the *Macavirus* genus of the subfamily *Gammaherpesvirinae* of the *Herpesviridae*. The best characterised of these are ovine herpesvirus-2 (OvHV-2) and alcelaphine herpesvirus-1 (AlHV-1), which are carried asymptomatically by sheep and wildebeest respectively. There is no apparent disease in these reservoir hosts, but when the virus is transmitted to susceptible animals, MCF often ensues [1]. MCF has a worldwide distribution wherever reservoir and susceptible animals co-habit. MCF is currently a serious problem in several parts of the world including: Indonesia, where cattle are infected by OvHV-2, North America, in farmed bison infected by OvHV-2 and in Eastern and Southern Africa where migrating wildebeest transmit AlHV-1 to cattle [1].

MCF in cattle caused by AlHV-1 and OvHV-2 is similar in clinical presentation with fever, inappetance, ocular opacity and oral epithelial lesions present in most cases [1-3]. Histologically, MCF is characterised by lymphocyte infiltration in multiple tissues, hyperplasia in lymph nodes and vasculitis most notably in the brain and kidneys [1-3]. The pathogenesis of MCF is an area of active study currently, and T cells (predominantly CD8+ T cells) accumulating within the tissues are virus-infected [4,5]. These cells may be responsible for mediating the tissue damage seen in MCF although this remains to be proven. The genomes of AlHV-1 and OvHV-2 have been sequenced showing that they are highly-related but distinct viruses [6,7].

Current control measures for MCF focus on keeping reservoir and susceptible species apart. This is not always possible, for example African pastoralists’ cattle that have been taken from the plains inhabited by wildebeest to upland regions to avoid MCF are then susceptible to the serious diseases of trypanosomosis and East Coast Fever [8,9]. There have been many attempts in the past to control MCF by vaccination although these have either failed or not been reproducible [10-14]. However, we have developed a vaccine strategy that is successful in preventing disease in cattle challenged with AlHV-1 by inducing a mucosal barrier of immunity in the oro-nasal pharyngeal region of cattle in response to intra-muscular immunisation in the upper neck with attenuated AlHV-1 in Emulsigen adjuvant [15]. However, the duration of immunity is short, peaking at around two months and lasting up to six months, and a boost immunisation is required to generate high titre virus-neutralising antibody that correlates with protection [15,16].

The aim of this study was to improve the magnitude and duration of protective immunity to AlHV-1 by using the TLR9 agonist unmethylated CpG oligodeoxynucleotide (CpG ODN) as an adjuvant. The TLRs are microbial pattern recognition receptors on or within epithelial and immune system cells, amongst others, that act as a first line of defence against infection [17]. TLRs working in concert or alone can influence the quality and duration of adaptive immune responses. Furthermore, AlHV-1, along with many other pathogens, has evidence of CpG suppression within its genome [6]. CPG ODN has been used successfully to enhance the magnitude or quality (Th1/Th2) of protective immunity to a range of pathogens and its duration in a variety of different species [18-20] including cattle [21-25]. Furthermore, there are various CpG ODN formulations that have different efficacies in any given species. The CpG ODN 2007 formulation used in this study has been proven effective in cattle previously [21-23].

Taking all this together, we have tested unmethylated CpG ODN as an adjuvant in our MCF vaccine formulation and compared it to emulsigen alone or with both adjuvants in combination. We used AlHV-1 challenge at 23 weeks after boost immunisation as this had previously achieved only 50% protection compared to challenge at 7–8 weeks (where > 90% protection has been recorded), and therefore should allow a measurement of the effect of CpG ODN on duration of immunity.

## Materials and methods

### Animals and study design

Clinically healthy and MCF seronegative male Friesian-Holstein calves, 6–7 months of age were used in the experiments. All animal experiments were approved by both the University of Nottingham and the Moredun Research Institute’s experiments and ethics committees and complied fully with the Home Office of Great Britain and Northern Ireland “Animals (Scientific Procedures) Act 1986”. For the study, calves were assigned randomly to four experimental groups and vaccinated intramuscularly in the upper neck as described in Table 1, with atAlHV-1 plus Emulsigen (Group 1, *n* = 9); atAlHV-1 plus Emulsigen and CpG (Group 2, *n* = 9); atAlHV-1 plus CpG (Group 3, *n* = 5); and Emulsigen plus CpG alone (Group 4, *n* = 5). Numbers were chosen based on power calculations using data from previous immunisation experiments [15,16] (see statistical analysis of data below). Intramuscular immunisation with atAlHV-1 in Emulsigen (20% v/v, MVP, Omaha, USA) was used in group 1 and group 2 calves for both prime and boost immunisations (separated by a period of one month) and for injection of atALHV-1 and CpGODN (group 3) and both adjuvants (group 4). Phosphorothioate-stabilized CpG ODN 2007 (TCGTCGTTGTCGTTTTGTCGTT) provided by Merial (Harlow, UK, purity > 90%) was included at 1 mg/injection in the immunisation of group 2 and 3 animals as this had previously been an effective adjuvant dose in cattle in various experimental vaccines [21-23].

**Table 1:** Experimental groups: clinical/histopathology analyses and serology

**Group**^**1**^	**Days after challenge**^**2**^	**Pathology/histology**^**3**^	**Viral DNA**^**4**^	**NS ab**^**5**^	**NS ab**	**Plas ab**^**6**^	**Plas ab**
				**Pre-chall**	**After chall**	**Pre-chall**	**After chall**
**Gp 1: Virus + Em**							
841	99	No MCF lesions	NDet	67	79	82	58
920	99	No MCF lesions	NDet	24	89	44	60
221	98	No MCF lesions	NDet	63	66	42	44
983	98	No MCF lesions	NDet	30	50	132	248
001	91	No MCF lesions	NDet	195	118	72	109
371	89	No MCF lesions	NDet	151	138	79	220
675	89	No MCF lesions	NDet	44	70	89	114
635	63	MCF Lesions: Lymphocytic inflammation and vasculitis. Pneumonia	32.6	35	96	47	449
101	42	Mild MCF lesions: Lymphocytic inflammation and vasculitis. Pneumonia	32.5	55	220	37	421
**Gp 2: Virus + Em +CpG**							
105	98	No MCF lesions	NDet	72	118	89	149
106	98	No MCF lesions	NDet	40	66	116	198
706	97	No MCF lesions	NDet	85	66	75	73
679	96	No MCF lesions	NDet	29	17	81	128
236	91	No MCF lesions	NDet	9	24	64	214
162	90	No MCF lesions	NDet	64	97	133	218
663*	90	Mild MCF lesions. Lymphocytic inflammation and vasculitis	33.6	6	234	51	931
400678	41	Mild MCF lesions: Lymphocytic inflammation and vasculitis. Mild pneumonia	37.5	0	300	60	324
065	35	MCF Lesions: Lymphocytic inflammation and vasculitis in multiple tissues. Pneumonia	27	0	408	26	299
**Gp 3: Virus + CpG**							
666	90	No MCF lesions	NDet	0	25	46	50
021	89	No MCF lesions	NDet	19	19	23	68
169	89	No MCF lesions	NDet	50	64	85	139
201027	65	Mild MCF lesions. Lymphocytic inflammation and vasculitis. Pneumonia	27.6	0	40	0	0
273	36	MCF Lesions. Lymphocytic inflammation and vasculitis. Pneumonia	26.2	0	0	0	220
**Gp 4: Adjuvant**							
658	88	No MCF lesions	NDet	0	0	0	0
101027	60	Mild MCF lesions. Lymphocytic inflammation	30.3	0	0	0	0
249	36	Mild MCF lesions. Lymphocytic inflammation and vasculitis in the brain and liver only	NDet	0	90	0	104
925	36	MCF Lesions. Lymphocytic inflammation and vasculitis. Pneumonia	24.7	2	74	0	0
986	27	MCF lesions: Lymphocytic vasculitis in multiple tissues. Pneumonia	30	0	15	0	81

^1^Em = emulsigen adjuvant, CpG = oligoCpG adjuvant.

^2^Time from challenge to euthanasia/post-mortem examination.

^3^Tissues in this analysis were: brain, lung, liver, kidney, prescapular lymph node, alimentary tract epithelium and mesenteric lymph node.

^4^Viral DNA was assayed in terminal blood samples by qPCR to give a specific and internally-controlled diagnostic test. Data are expressed as Ct values – the cycle number at which specific amplification was first detected. Moderate/severe MCF usually has values in the range 21–29. NDet = specific amplification of AlHV-1 DNA not detected (but control actin-specific PCR was detected). *This animal had histological evidence of infection.

^5^Nasal secretion antibody (NS ab) titres pre-challenge and after challenge with virus.

^6^Plasma antibody (Plas ab) titres pre-challenge and after challenge with virus.

Animals in groups 1, 2 and 3 received the same dose of vaccine (1 mL of 10^7^ TCID_50_ per mL) for both prime and boost, given intramuscularly and in groups 1 and 2 contained 20%(v/v) Emulsigen. Control animals in group 4 received virus-free culture medium with Emulsigen prepared and administered in the same way. Animals in all groups were challenged 23 weeks after boost with 10 mL of 10^3.8^ TCID_50_/mL of virulent AlHV-1 given intranasally. For mild MCF lesions, Viral DNA in the tissues can be below detection level.

All animals were challenged with 10 mL of 10^3.8^ TCID_50_/mL virulent AlHV-1 given intranasally by spraying 5 mL per nostril at 23 weeks after boost immunisation (week 27 of the experiment, Table 1 legend). In all experiments, uncoagulated blood and nasal secretions were collected from all animals immediately prior to primary immunisation and weekly thereafter as described previously [15,16]. After virus challenge, animal rectal temperatures were measured daily and clinical scoring was performed daily after the onset of pyrexia (defined as rectal temperature > 40 °C). Clinical scoring, based on temperature, body condition, ocular and nasal lesions, was used to ensure that all animals were euthanized, with an overdose of intravenous sodium pentobarbitone, at the onset of moderate clinical signs [16].

At autopsy, the following tissues were collected: brain, buccal mucosa, rumen, reticulum, liver, kidney, lung, prescapular lymph node, mesenteric lymph node (MLN) and blood. Pieces of each tissue (except blood) were fixed in (a) 10% formal saline (b) 4% paraformaldehyde (c) zinc salts fixative [15,16] before processing routinely and embedding in paraffin wax. Live cell suspensions of MLN were cryopreserved under liquid nitrogen.

For histological analysis, 4 μm sections of formalin-fixed tissues were cut, mounted on glass microscope slides and stained with haematoxylin and eosin. In addition, total DNA was prepared from blood buffy coat cells for PCR detection of viral DNA.

### Virus

The strains of AlHV-1 used for vaccination and challenge were as described previously [15,16]. Briefly, the virulent C500 strain virus (virAlHV-1) was collected from cultures of bovine turbinate (BT) cells infected with a lymphoid cell suspension derived from pooled lymphoid tissue from rabbits infected with AlHV-1 C500 that had developed MCF. Infected BT cell cultures were passaged onto fresh BT cells by a 1:4 split four times at peak cytopathic effect (approximately weekly) after which virulent virus was harvested from culture supernatants and cells following three rounds of freeze-thaw treatment of the cells. Cell-free virus supernatant was stored at −80 °C as 1 mL aliquots of pooled virus in batches and representative aliquots of each batch were titrated to allow calculation of the appropriate challenge dose. Titration measured 50% tissue-culture-infectious dose (TCID_50_) as described previously [15,16].

The attenuated AlHV-1 C500 strain (atAlHV-1) at passage > 1000 was used as the source of virus for immunisation [15,16]. This cell-free virus was obtained from BT cell culture supernatants, clarified by centrifugation and stored in batches at −80 °C until required. Representative aliquots of atAlHV-1 were titrated as described for virulent AlHV-1. The virus vaccine dose for both prime and boost comprised 1 mL of 10^7^ TCID_50_ per mL atAlHV-1 with adjuvant(s) as described above.

### Detection of viral DNA

Viral DNA was assayed in pure genomic DNA samples extracted from blood buffy coat cells by quantitative (q)PCR as described previously [15,16]. Briefly, ~50 ng of total DNA was assayed simultaneously for the presence of AlHV-1 and genomic actin sequences by duplex real-time PCR analysis. Each 20 μL assay contained 900 μM of each AlHV-1 forward and reverse primer (AlHV1-F, AlHV1-R) and 250 μM of the fluorogenic probe FAM-AlHV1; 450 μM of the genomic actin primer (gACT-F: 5’-CAC CTT CCA GCA GAT GTG GA-3’; gACT-R: 5’-CTA GAA GCA TTT GCG GTG GAC) and 125 μM of the fluorogenic minor-groove binding (mgb) probe VIC-gACT (5’-VIC-AGC AAG CAG GAG TAC G-mgb) [26]; and real-time PCR reagents containing Platinum Taq polymerase and ROX control dye (Invitrogen (Life Technologies), Paisley, UK). All assays were conducted using standard conditions on ABI 7000 or 7500 sequence detection systems (Applied Biosystems (Life technologies), Paisley, UK).

### Antibody detection by ELISA and the virus-neutralising antibody test

The antibody ELISA was used to detect humoral virus-specific antibody titres in blood plasma samples and nasal secretions as described previously [16]. An AlHV-1-negative serum at 1:500 dilution was also included with each test sample series.

ELISA values (difference between means of positive and negative antigen wells for each sample dilution) were used to calculate a relative titre for each test sample, based on the standard curve produced from the positive control serum wells on the same plate. Test samples with ELISA values outside the range found for the positive control serum were discarded. Standard pools of NS and plasma were used to prepare serial dilutions (NS 1:20–1:2560 and plasma 1:200–1:6400) that were used on each test plate. ELISA values from these standards were used to generate a best fit polynomial standard curve (y = ax^2^ + bx + c) for each plate with R^2^ value greater than 0.98. Relative titres of unknown samples were calculated from the plate standard curve using NS samples at a dilution of 1:160 and plasma at 1:500.

The virus neutralisation test was based upon 50% inhibition of AlHV-1-induced cytopathic effect (CPE) in BT cells by dilutions of blood plasma or nasal secretion fluid as described [15,16]. Assays were carried out in 96 well tissue culture plates with BT cells at greater than 80% confluence. All assays used a high titre bovine anti-AlHV-1 serum as a standard and included non-specific toxicity-control wells containing sample and cells without virus.

### ELISPOT and ELISA for interferon-γ and interleukin-4 detection

An ELISPOT test to quantify IFN-γ release from bovine PBMCs was carried out using Millipore Multiscreen HTS IP 0.45 μm ELISPOT plates according to the manufacturer’s instructions (Millipore, Watford, UK). Wells were coated with 100 μL (5 μg/mL) anti bovine-IFN-γ antibody (clone CC330, AbD Serotec, Oxford, UK) and blocked with Iscove’s modified Dulbecco’s medium (IMDM) containing 10% FCS. PBMCs were added at 1 × 10^5^ per well in medium. Viral antigen, previously prepared using 3-[(3-cholamidopropyl) dimethylammonio]-1-propanesulfonate (CHAPS) detergent from the C500 strain of AlHV1 was purified using Amicon Ultra-0.5 μm Centrifugal Filter Units (Millipore, Oxford, UK) and quantified using a Bradford protein assay (Life Science, Hemel Hemstead, UK) and added to wells in triplicate at 0.2 μg per well. A combination of 0.1 ng phorbol 12-myristate 13-acetate (PMA, Sigma-Aldrich, Dorset, UK) and 50 ng Ionomycin (Sigma-Aldrich, Dorset, UK) per 10^5^ cells in each of two wells were used to stimulate IFN-γ production as a positive control. PBMCs in medium comprised the negative control. Cells were incubated for 72 h at 37 °C in a humidified 5% CO_2_ in air tissue culture incubator. Biotinylated anti-bovine IFN-γ (clone CC302, AbD Serotec, Oxford, UK) was used as the detection mAb at 4 μg/mL (100 μL/well). ExtrAvidin Alkaline Phosphatase and Sigma fast BCIP/NBT substrate (Sigma-Aldrich, Dorset, UK) was utilised for spot development. Spots were then counted using an ELISPOT reader (AID GmbH, Oxford Biosystems, Oxford, UK) and averages taken from antigen exposed triplicates and negative control triplicates, the difference gives spot forming cells (SFC).

Supernatant samples (50 μL) collected after 72 h incubation of the PBMCs with viral antigen were tested using the Thermo Scientific Bovine IL-4 ELISA Kit (Thermo-Fisher Scientific, Loughborough, UK. Product# ESS0031).

### Pathology and histopathological analyses

Clinical MCF was diagnosed by clinical signs and confirmed by histopathological lesions and the detection of virus DNA in terminal blood samples. In clinically normal animals, infection was deemed to have occurred if there was histological evidence of lesions and viral DNA in the tissues. Clinical signs included fever, nasal and ocular discharge, conjunctivitis and development of corneal opacity. The use of a clinical scoring system ensured that all animals with MCF were euthanized at a similar stage of the disease. All surviving animals were euthanized around 13 weeks after pathogenic virus challenge. Clinical scores and histopathology were recorded. MCF histopathology in brain, kidney, liver, lung, lymph nodes and alimentary tract epithelium was examined and scored for the frequency of lesions consistent with MCF. The pathology of each case was summarised as MCF (tissues contained significant numbers of lesions consistent with a diagnosis of MCF); mild (a small number of lesions, without extensive infiltration of lymphocytes, which may or may not be associated with clinical disease); or negative (no significant lesions observed). A positive test for AlHV-1 DNA in the blood on the day of autopsy, in combination with typical MCF histopathology, was taken as a definitive diagnosis of either clinical or non-clinical MCF.

### Statistical analysis of data

For the survival plot comparisons, a log rank test was used. For the antibody titre and ELISPOT data comparisons, the Mann Whitney test and Student’s *t*-test were used. For animal group sizes, a power calculation based on Fisher’s test was applied [27].

Normally the group size is defined as that which gives an 80% chance of detecting a difference between treatment groups. We assume (based on experimental data) that 93% of unvaccinated animals would get MCF (in the virus challenge group) and that we want to detect protection at a level of 50% or better. We know from previous experiments that 6 months after immunisation boost there will be a 50:50 ratio of survivors to cattle that succumb to MCF. We estimate that there will be a 80% chance of detecting a significant difference at a two sided 0.8 significance level. This equates to using at least 8 cattle for groups 1 and 2. We used 9. For group 3 there is no published evidence in cattle that immunogen with CpGODN alone induces a significant protective response compared to protection positive controls (16–20), so for this and group 4 animals group sizes were reduced to 5 but still retained statistical power. Previous work had shown that atALHV-1 alone did not induce an MCF protective response in cattle after virus challenge (unpublished results).

## Results

### The effect of CpG ODN adjuvant on survival rates in vaccinated cattle

**Figure 1:**
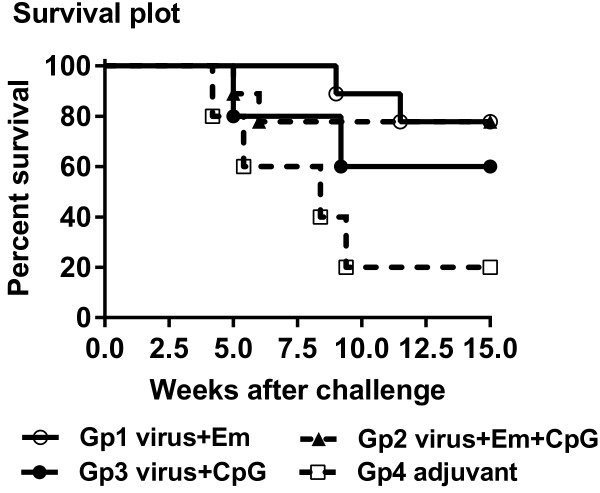
**Survival plot for each of the four groups of cattle.** Kaplan Maier survival plot. Group 1: 9 cattle immunised and boosted with atAlHV-1 (virus) along with Emulsigen (Em) adjuvant. Group 2: 9 cattle immunised and boosted as in group 1 but including CpG ODN (CpG) as well as Emulsigen; Group 3: 5 cattle immunised and boosted with atAlHV-1 (virus) and CpG ODN. Group 4: 5 cattle injected with adjuvant only. *P* = 0.02 for group 1 compared to group 4. *P* = 0.04 for group 2 compared to group 4. Groups 3 and 4 were not significantly different (*p* = 0.24).

Animal details, including time to euthanasia for animals developing MCF, histopathological findings and detection of viral DNA in blood samples, are detailed in Table 1, while survival (protection from clinical MCF) is plotted by group in Figure 1. Two of nine animals receiving the standard vaccine formulation (Group 1) developed MCF after challenge, compared with development of MCF in four of five animals that received only Emulsigen (Group 4), showing that the vaccination and challenge protocol employed here was effective. Immunisation using the CpG ODN adjuvant with atAlHV-1 in the immunisation protocol (Group 3) did not induce significant protective immunity against virulent AlHV-1 challenge (Figure 1). Although 3 of 5 animals in group 3 were protected, this was not significant (*P =* 0.24) compared to the control group 4 animals (adjuvant only group receiving virus challenge). When CpG ODN was used in combination with emulsigen and AtAlHV-1 (Group 2), there was no increase in survival rate/protection compared to using emulsigen alone with atAlHV-1 (Group 1). Both group 1 and group 2 cattle were significantly protected (with respect to survival) over the duration of the experiment when compared to the infection control animals in group 4 (*P =* 0.02 and *P =* 0.04, respectively). Postmortem analysis indicated no evidence of infection in any of the protected cattle except one group 2 animal (663). Table 1 shows the clinical and histopathological findings of the individual animals in each group. Clinical presentation for cattle with MCF was: inappetance, fever (>40 °C for two or more consecutive days) and ocular and nasal discharge with ocular opacity in most cases. Histologically, in MCF-affected animals the lesions consisted of interstitial and/or perivascular lymphocyte accumulations in non-lymphoid organs (kidney, liver, lung and brain), and hyperplasia and some architecture disruption in the lymphoid tissues. In both types of tissues there was evidence of vasculitis. All of this is typical of MCF in cattle. Mild MCF presentations were probably due to euthanasia of the animals at mild to moderate rather than moderate to severe clinical scores in accordance with Home Office (UK) ASPA MCF end point criteria. This could well have been responsible for the low titres of AlHV-1 DNA in the blood cells in some animals, for example in group 4 (Table 1). This qPCR analysis of AlHV-1 DNA in terminal blood samples confirmed the clinical and histopathological diagnosis of MCF (Table 1). The survivor of clinical disease in group 2 (663), showed histological evidence of very mild infection, and was therefore considered infected but clinically free of disease. In animals that showed evidence of clinical MCF, lesions suggestive of bacterial pneumonia were present also in most of them (8 of 11, Table 1). 663 was not one of these. Of the 17 animals protected from MCF, there were none that presented with evidence of pneumonia at autopsy.

### Antibody responses to immunisation and virus challenge

**Figure 2:**
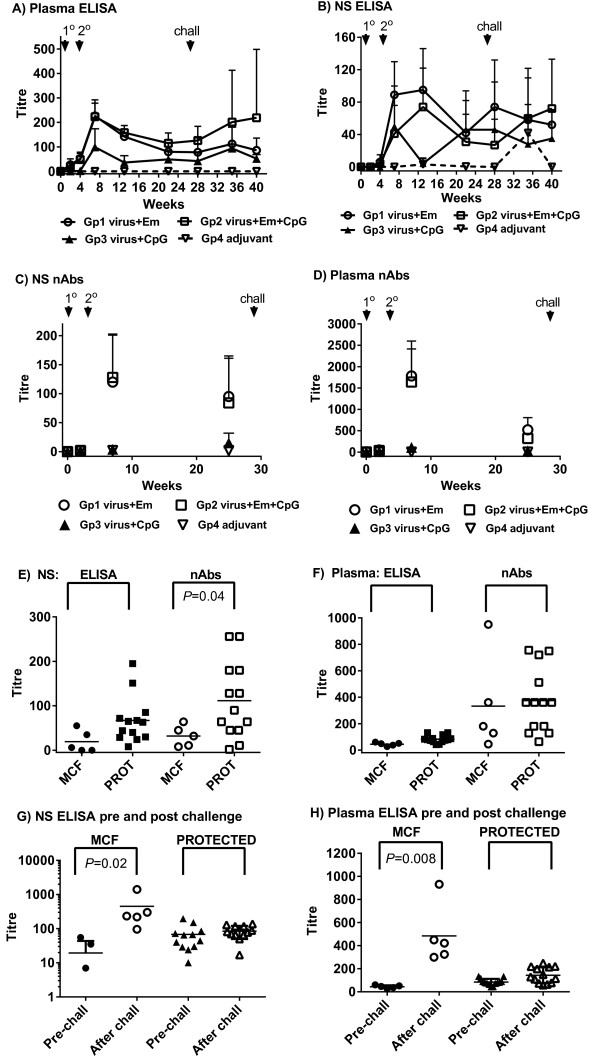
**Virus-specific antibody responses. (A, B)** Virus-specific antibody titres in each of the 4 groups of cattle detected by ELISA. Group mean titres and standard deviation of the mean (SD, bars) are shown for each time point. Arrows show timing of primary (1°) and boost (2°) immunisations and virus challenge on week 27 (chall). **(A)** Blood plasma antibody; **(B)** antibody in nasal secretions (NS). **(C, D)** Virus neutralising antibody titres in each of the 4 groups of cattle. These were performed on samples prior to immunisation, at week 7 (3 weeks after booster immunisation) and on week 26, 1 week prior to virus challenge. Group mean titres and SD are shown for each time point; **(C)** Neutralising antibody titres in nasal secretions; **(D)** Neutralising antibody titres in blood plasma; **(E, F)** Comparison of total virus-specific antibody titres and virus-neutralising antibody titres in group 1 and 2 at week 26, just prior to virus challenge, in cattle that subsequently developed MCF or were protected (PROT); **(E)** Comparison in nasal secretions; **(F)** Comparison in blood plasma. *P* values for any significant comparisons are shown in the figures. **(G, H)** Comparison of total virus-specific antibody titres on week 26, just prior to virus challenge (pre-chall) and after challenge (taking the highest titre of the various time points after challenge) in animals that developed clinical MCF compared to those that were protected (PROT). (G) Nasal secretion antibody comparison. **(H)** Blood plasma antibody comparison. *P* values for any significant comparisons are shown in the figures.

Following the primary immunisation, total virus-specific antibody in both plasma and nasal secretions were low, but increased after a booster immunisation (Figure 2A, 2B) in groups 1–3. However, group 3 (CpG ODN adjuvant plus atAlHV-1) animals had lower mean titres than groups 1 and 2 at most time points and this was significant on week 13 (week 9 after boost immunisation) in both the plasma and nasal secretion samples (Figure 2A, 2B). The peak antibody responses occurred around week 7 (3 weeks after boost) in plasma (Figure 2A) and between weeks 7 and 13 for groups 1 and 2 animals in nasal secretions (Figure 2B), after which titres then declined. Whereas virus-neutralising antibodies were present in the plasma and nasal secretions of group 1 and group 2 cattle, these were absent or at very low titre in the group 3 animals (CpG ODN adjuvant plus atAlHV-1). Titres were similar in groups 1 and 2 animals, where the group 2 animals were immunised with atAlHV-1 along with both emulsigen and CpG ODN adjuvant (Figure 2C, 2D). Total virus-specific antibody titres in nasal secretions and plasma were measured after AlHV-1 challenge, and there was a wide distribution of values, particularly in group 1 and group 2 animals (Figure 2A, 2B).

### Antibody responses associated with protection

To determine whether virus specific antibody titres (either total or virus neutralising antibodies) at around the time of virus challenge were associated with protection, they were analysed in vaccinated animals from groups 1 and 2 in which protection from MCF was observed. Titres from cattle that succumbed to MCF (*n* = 5) were compared to those that did not (*n* = 13). This included the clinically normal animal in group 2 that had histological evidence of AlHV-1 infection. Figure 2E shows that, although titre distributions were large, significantly higher neutralising antibody titres in nasal secretions at around the time of virus challenge (week 27) were associated with animals that were protected compared to those that succumbed to, or had evidence of, MCF (*P* = 0.04). Plasma neutralising antibodies did not correlate with protection, nor did total virus-specific antibodies in either plasma or nasal secretions (Figure 2E, 2F). To further explore the variation in total virus-specific antibody titres in animals after virus challenge (Figure 2A, 2B), the titres were compared before (week 26) and after challenge in nasal secretions and plasma in animals with MCF or those that were protected (Figure 2G, 2H). The highest titre for each animal sample was selected from within the various time points after challenge to make the comparison more robust. There was a significantly higher mean antibody response in the nasal secretions and plasma samples from MCF affected animals in groups 1 and 2 after challenge compared to before challenge (Titres of 19 ± 24 for nasal secretion antibodies pre-challenge and 252 ± 114 after challenge; titres of 44 ± 13 for plasma antibodies pre-challenge and 484 ± 257 after challenge) whereas neither nasal secretion titres nor plasma titres were different before and after challenge in the protected animals.

### The effect of the different adjuvants on IFN-γ- and interleukin-4 secreting cells

**Figure 3:**
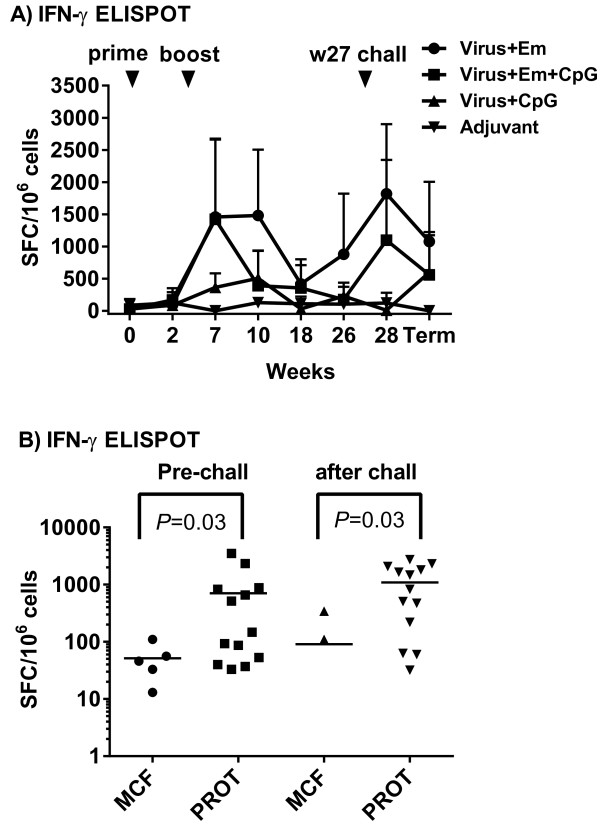
**IFN-γ secreting PBMC frequencies. (A, B)** IFN-γ-secreting cells expressed as spot-forming cells (SFC) per 10^6^ PBMC. Blood samples from weeks 0, 2, 7 (3 weeks after booster immunisation), 10, 18 and 26 (1 week prior to virus challenge- Pre-chall) were taken, as well as week 28 and the terminal blood sample prior to euthanasia (Term sample) in both the MCF and protected animals. **A)** Analysis of all animals in each group shown as group means and standard deviation of the mean (SD). **(B)** IFN-γ-secreting cell frequencies in individual animals (displayed as a scatter plot) comparing animals that developed MCF or were protected (PROT). Significant responses were recorded for the pre-challenge and after challenge terminal samples only. Note there were 3 terminal blood PBMC samples with 0 SFC in the MCF group and these do not show on the log scale used.

The secretion of the key Th1 and Th2 cytokines, interferon-γ (IFN-γ) and interleukin-4 (IL-4) respectively, was assayed in PBMC isolated from uncoagulated blood collected at a range of time-points during the trial (Figure 3). Group 1 animals showed the highest group mean value for IFN-γ for each time point, starting at 3 weeks after immunisation boost (week 7 of the experiment), whereas group 2 animals showed an initial good response at this time that subsequently declined thereafter up to virus challenge at week 30 of the experiment (Figure 3A). Group 3 animal PBMCs contained IFN-γ secreting cells notably on weeks 7 and 10. Wide variation was seen in the data for each group at each time point (Figure 3A), such that there was no significant differences between groups 1–3 comparisons at any individual time point. After virus challenge the mean frequency of IFN-γ-secreting cells in PBMC increased in groups 1 and 3 animals compared to week 26 pre-challenge levels, although this was not significant. A comparison of IFN-γ-secreting cells in blood samples taken just prior to virus challenge (week 26) and in the terminal blood samples prior to euthanasia indicated that animals protected against MCF exhibited significantly higher numbers of cells producing IFN-γ (after in vitro stimulation with virus antigen) than those that succumbed to MCF (Figure 3B, *P* = 0.03 for an analysis on each day).

IL-4 was not detected in any of the samples (results not shown).

## Discussion

In this study, we wished to determine whether the TLR9 agonist CpG ODN used with atAlHV-1 alone or in combination with Emulsigen and atAlHV-1 could improve the magnitude and duration of protective immunity afforded by our vaccine against AlHV-1 associated MCF. We show that the inclusion of CpGODN as an adjuvant was not effective in this regard, nor did it affect the degree of disease protection obtained with atALHV-1 and emulsigen alone. Previous studies using a similar immunisation regime involving CpGODN in BoHV-1 immunisation [22,23] had shown that CpG ODN included with emulsigen increased the duration of immunity and also enhanced a IFN-γ response in T cells (Th1-like response) as evidenced by increased cellular production of IFN-γ compared to Th2 cytokine responses (IL-4). Furthermore, in other studies where CpG ODN was included with a “traditional” well-established adjuvant (e.g. montanide) and either nominal antigen (hen egg lysozyme [25]) *Mycoplasma bovis*[21] or foot and mouth disease virus (FMDV) [24]) an increase in the duration of immunity and also an enhanced Th1 response as evidenced by increased cellular production of IFN-γ compared to Th2 cytokine responses was recorded. In contrast, in this study, the CpG ODN used in atAlHV-1 vaccine formulations on its own or in combinations with Emulsigen as an adjuvant did not significantly alter the period of protective immunity or enhance the magnitude of the immune response parameters measured over that achieved with Emulsigen alone with atAlHV-1 (Figure 1, Table 1, Figure 2). Total virus-specific antibody titres and virus-neutralising antibody titres in blood plasma and nasal secretions were not enhanced by the inclusion of CpG ODN, and neither was the frequency of IFN-γ expressing blood cells restimulated in vitro. IL-4 secretion by blood leukocytes was not detected. The reason for the difference between our study and the others may be that the combination of atALHV-1 with emulsigen is already maximally efficient at inducing an IFN-γ response and this cannot be improved upon by the inclusion of CPGODN, leading to an increased duration of immunity (assuming there is a correlation). In aged pigs given pseudorabies vaccine, a suboptimal Th1 response was restored when CpGODN was used as an adjuvant (27). Comparing our study with the BoHV-1 subunit ones [22,23], which follow a very similar immunisation regime, would indicate that the principal difference is in the nature of the immunising antigens. AtALHV-1 virions were used in our study compared to BoHV-1 subunit proteins in the other studies [22,23]. This is worthy of further study.

In this study, the use of CPG ODN on its own with atAlHV-1 was not effective in inducing protection to clinical MCF, and this is also the case in the other studies in cattle where CpGODN was used as an adjuvant on its own [21-25] and in pigs with FMDV vaccination [28]. We have previously shown that the atAlHV-1 remains viable when mixed with Emulsigen adjuvant but no evidence of virus replication or persistence after prime and boost vaccination has ever been detected [15], and unpublished result.

In spite of the lack of vaccine improvement by CpG ODN, which in itself is an important result, there were several other novel, aspects to the study. We have quantitatively-confirmed our previous observation [16] that virus neutralising antibody responses in nasal secretions just prior to virus challenge correlated with protection (*P =* 0.04*,* Figure 2E, 2F) in group 1 and group 2 animals. Neither plasma antibody (total virus-specific or virus-neutralising antibody) correlated with protection, nor did total virus-specific antibody in nasal secretions (Figure 2E, 2F). This supports the hypothesis that a mucosal barrier with neutralising antibody is important to inhibit AlHV-1 infection to a point where disease is prevented. Notably, despite 3 of 5 animals being protected in group 3 (CpG-ODN plus atALHV-1 immunised animals), CpG-ODN as adjuvant on its own induced very low or no virus-neutralising antibody responses (Figure 2C, 2D). However, these animals exhibited moderate total virus-specific antibody responses in both plasma or nasal secretions (Figure 2A, 2B).

This suggests that immune mechanisms other than virus-neutralising antibody may influence protection from virAlHV challenge. Unfortunately, despite thorough investigation of methods that could be used for the detection of cytotoxic T cells (CTLs) in these experiments, no CTL assay that we developed was robust enough (reproducibility, sensitivity) to address this aspect of immune responsiveness in this experiment.

After challenge with virus, animals that developed MCF showed a significant increase in virus-specific antibody titres (before succumbing to disease) compared to levels prior to challenge, whereas those that were protected did not (Figure 2G, 2H). This increase was more pronounced in the plasma of MCF-affected animals than in their nasal secretions (Titres of 19 ± 24 for nasal secretion antibodies pre-challenge and 252 ± 114 after challenge; titres of 44 ± 13 for plasma antibodies pre-challenge and 484 ± 257 after challenge). This further reinforces the view that protected animals prevent virus from entering the host by the oro-nasal route and thereby stimulating a systemic antibody response. Furthermore, the more pronounced effect of challenge on circulating antibody levels in MCF animals supports this, as virus establishing systemically would be expected to stimulate an increase in plasma antibody titres. In spite of stimulating a high titre antibody response, all but one of the animals succumbed to disease, so this response is not protective. In the clinically-normal animal that had evidence of infection post-mortem, there was also evidence of an antibody response indicating a response to infection (Table 1). The fact that this animal survived beyond the time that all others had succumbed to MCF might indicate that whereas vaccination protects against disease it does not protect against infection in all cases, or vaccination induced a delay in onset. In previous experiments testing our vaccine, animals succumbed to MCF within the time to euthanasia set in this study, with one group of survivors showing no signs of MCF up to a year after challenge (15,16).The frequency of IFN-γ producing cells (after in vitro stimulation of PBMC in culture with virus antigen) was high in group 1 and group 2 animals, indicating a good response to immunisation and boost with emulsigen with atAlHV-1 (Figure 3A) or emulsigen and CpG ODN with atAlHV-1. This was confirmed by the association of a significantly-higher frequency of IFN-γ secreting cells in protected animals compared to those that succumbed to MCF in groups 1 and 2, both in week 26 pre-challenge samples and in PBMC samples taken prior to euthanasia (Figure 3B). The inclusion of CpG ODN with emulsigen in the vaccine formulation (group 2 animals) did not enhance the response compared to that supported by emulsigen alone (Figure 3A), but with CpG ODN on its own with atAlHV-1 (group 3) there was a small effect on IFN-γ secreting cells. The lack of an augmentation of IFN-γ producing cells by CpG ODN in group 2 animals is different to other studies, where the adjuvant had an effect in this respect (21–24).

IL-4 was not detected in PBMC cultures. IL-4 is a key indicator of Th2 responses but levels recorded in cattle are usually low in the absence of a strong Th2 response [29]. The lack of detection herein does not rule out Th2 involvement, but the frequency of IFN-γ expressing cells does suggest a good Th1 response.

Finally, an interesting observation is that animals with clinical MCF also showed evidence of bacterial pneumonia at autopsy, whereas animals with no evidence of clinical disease did not exhibit the pneumonia. This raises the possibility that MCF might predispose cattle to pneumonia.

### Conclusion

In conclusion, CpG ODN did not alter the quality, magnitude or duration of immunity in cattle immunised against AlHV-1 MCF when used in combination with emulsigen which is in contrast to other studies in cattle showing improved immunity in immunisation regimes to other pathogens [21-24]. It may be that further adjuvants are necessary, such as the use of host defense peptide and phosphosphazine in combination with CpGODN [25]. The production of a mucosal barrier of virus-neutralising antibody at the oro-nasal transmission entry site of AlHV-1 to prevent infection and the establishment of disease is successful in our prime-boost immunisation strategy, but peaks at approximately two months after booster immunisation. Mucosal immune responses are tightly regulated and finding a way of improving the magnitude and duration of protection against AlHV-1 MCF remains a challenge.

## Competing interests

The authors declare that they have no competing interests.

## Authors’ contributions

Experimental design and planning: DMH, NP, GCR, DG, HT, KB; animal experiments DMH, NP, GCR, DG, HT, KB; pathology analysis DG, HT, MD; serological and immunological analysis DD, NP, DMH, DG; data processing and statistical analysis DD, DG, NP, DMH, GCR; drafting of the manuscript DMH, MD, GCR. All authors read and approved the manuscript.
